# Gastric collision tumour with probable ectopic pancreatic origin

**DOI:** 10.3332/ecancer.2022.1410

**Published:** 2022-06-13

**Authors:** Juan Francisco Olivos Gonzáles, Rodrigo Arroyo-Gárate, Miguel Angel Leon Estrella, Gustavo Cerrillo, Stefanie Campos Medinae

**Affiliations:** 1 Instituto Nacional de Enfermedades Neoplásicas, Surquillo 15038, Peru; 2 Instituto Regional de Enfermedades Neoplásicas del Centro, Concepción 12126, Peru; 3 Hospital Nacional Dos de Mayo, Cercado de Lima 15003, Peru; 4 Department of Anatomical Pathology, Hospital Nacional Dos de Mayo, Cercado de Lima 15003, Peru; 5 Universidad Nacional Mayor de San Marcos, Lima 15081, Peru; 6 Universidad San Martín de Porres, Lima 15024, Peru; a https://orcid.org/0000-0002-0559-0295; b https://orcid.org/0000-0002-8186-0859; c https://orcid.org/0000-0001-5026-7940; d https://orcid.org/0000-0001-6209-0214; e https://orcid.org/0000-0003-1519-1738

**Keywords:** neoplasms, stomach neoplasms, pancreatic neoplasms

## Abstract

**Introduction:**

Mixed histology tumours are rarely found in the stomach. Of these, collision tumours are mainly composed of adenocarcinomas and sarcomas or lymphomas. This is the seventh case reported in the literature of an acinar cell carcinoma arising from an ectopic pancreas located in the stomach and the first described within a collision tumour.

**Clinical case:**

We present the case of a 58-year-old female patient diagnosed with gastric cancer who, after undergoing a total gastrectomy, presented with a pathology report describing findings compatible with gastric collision tumour with components of tubular adenocarcinoma and acinar cell carcinoma of probable pancreatic ectopic aetiology.

**Discussion:**

At the beginning of the 20th century, collision tumours were rarely described. Those located in the stomach are an infrequent pathology and are rarely diagnosed preoperatively. A collision tumour is composed of two independent neoplastic tissue with tumour areas separated in two different histological patterns and, in case of metastasis, this separation must also be clearly identified. There are different theories about its carcinogenesis and the debate regarding the ideal treatment is still ongoing.

**Conclusion:**

This is the first report of a malignant gastric tumour with probable heterotopic pancreatic origin that collides with gastric adenocarcinoma.

## Introduction

Mixed histology tumours rarely occur in the stomach or at the gastroesophageal junction. Within this category are adenosquamous carcinomas, mucoepidermoid carcinomas and collision tumours. Regarding the latter, the vast majority of reported cases are collisions between adenocarcinoma and sarcoma or lymphoma [1].

The description of collision tumours dates back to the beginning of the 20th century and defined as: ‘malignant tumours that originated in two different locations and, during their growth, invade each other, especially in their border areas’ [2]. They have been reported multiple times in different locations, such as the cervix [3], lungs [4], anorectal junction [5], oral cavity [6], liver [7, 10], bladder [8] and skin [9].

The aim of this article is to present the case of a gastric collision tumour composed of a tubular adenocarcinoma and an acinar cell carcinoma probably arising from an ectopic pancreas. The latter is infrequently reported, being this the seventh case described in the literature of acinar cell carcinoma arising from a heterotopic pancreas located in the stomach [28] and the first described within a collision tumour.

## Clinical case

This is a case of a 58-year-old woman, housewife, with a history of osteoporosis diagnosed 4 years before admission, treated with calcium supplements and bisphosphonates. Also, she has a self-reported family history of leukaemia (her father died of leukaemia, does not specify the type). She denies smoking, alcohol consumption or use of illicit drugs.

The patient was admitted to the Emergency Department on 4 April 2018, with stable vital signs and reported cramp-like epigastric pain spreading to the mid-abdomen over the preceding 3 weeks, associated to mild abdominal distension, hyporexia, weight loss (approximately 7 kg), nausea, haematemesis and melaena.

On physical examination, she had a regular general condition, mildly nourished and pale skin and mucosas. The patient had globular abdomen with normal bowel sounds; no abdominal distension; and experienced mild pain to deep palpation in the epigastrium and mid-abdominal area. No mass was found. On digital rectal examination, she had a normal anal sphincter tone, empty rectal ampulla and no blood was observed on the gloved finger.

The differential diagnosis at the time was non-active upper gastrointestinal (GI) bleeding. Initial tests carried out included blood testing that showed microcytic hypochromic anaemia (haemoglobin = 7 g/dL, mean corpuscular volume = 58 fL, mean corpuscular haemoglobin = 16.3 pg) and leucocytosis (*white blood count* = 11.8/mm^3^ – 74% neutrophils, 16% lymphocytes, no bands). C-reactive protein was 23.8 mg/L. Treatment was as follows: nothing by mouth, transfusion of 1 unit of packed RBCs, proton pump inhibitor (Omeprazole), intravenous hydration and interconsultation to Gastroenterology. The patient was hospitalised.

Upper GI endoscopy was performed on 11 April 2018 with the following findings: at the subcardial level (between the greater curvature and posterior wall), a proliferative lesion of 4 cm in diameter was observed; very friable when touched by the equipment; soft when biopsies were taken. Diagnosis was advanced gastric malignant neoplasm – Bormann I.

After all studies were completed, the patient underwent surgery by the upper GI surgery team. During the procedure, the following findings were described:

- A 10 x 18 cm solid tumour in the body and subcardial region, radiating to the posterior wall and the lesser curvature, involving the serosa and infiltrating the pancreatic body and celiac trunk.
- Multiple perigastric lymph nodes.

A total gastrectomy + Roux-en-Y end-to-end oesophagojejunal anastomosis was performed.

The patient had a favourable PO evolution and was discharged from the surgery department.

**Figure 1.**
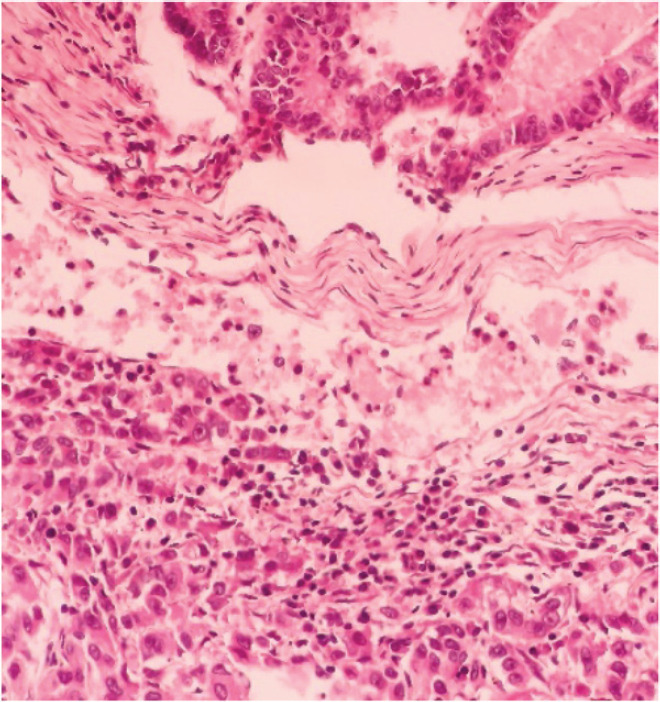
Intestinal pattern neoplastic component (upper) and pseudo-acinar pattern component (lower) separated by vascularised connective tissue.

**Figure 2.**
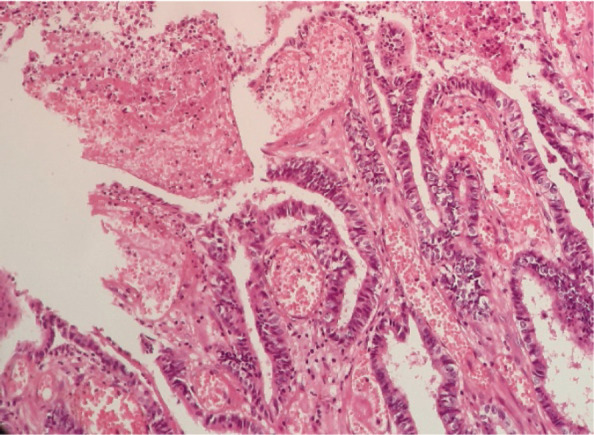
Microscopic examination (H&E stain). Extensive malignant neoplastic proliferation in the intestinal pattern component. The columnar epithelial lineage stands out, as well as the areas of coagulative necrosis.

**Figure 3.**
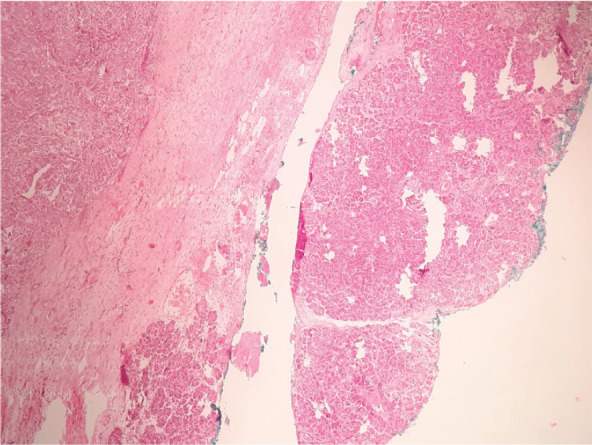
Heterotopic pancreas adjacent to gastric wall infiltrated by acinar cell carcinoma.

**Figure 4.**
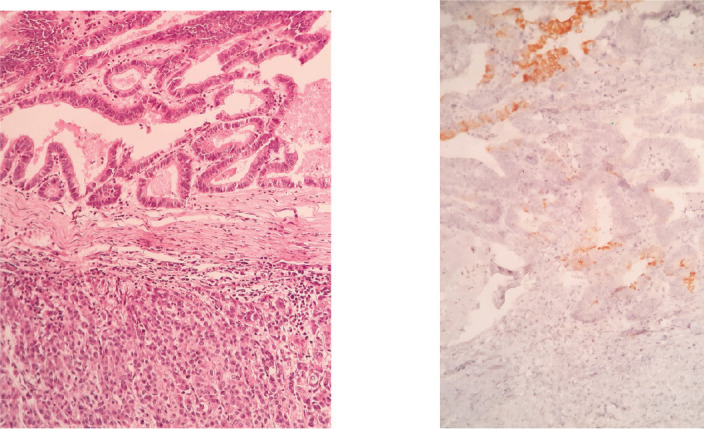
H&E stain (left) and Cytokeratin-20 (right).

**Figure 5.**
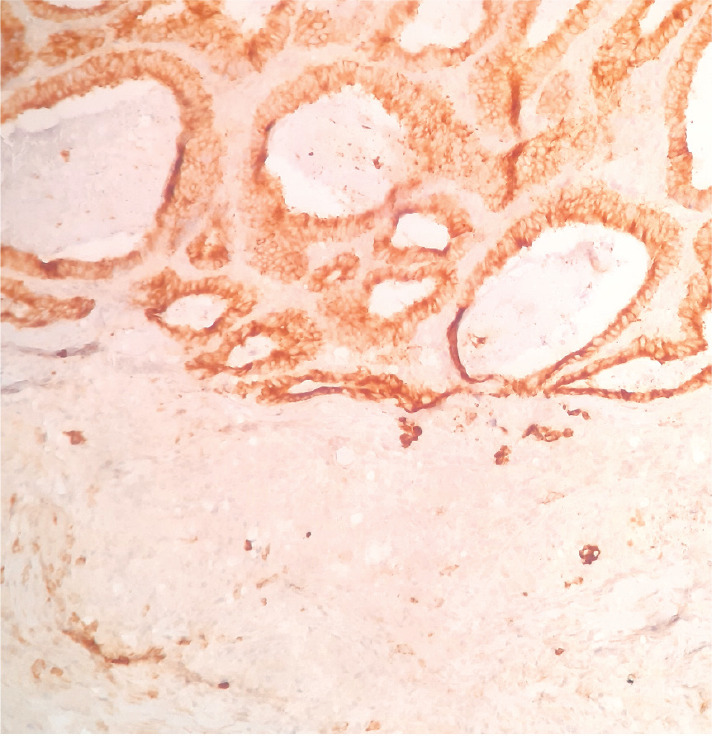
Plasma membrane antigen. This marker is generally distributed in many epithelial neoplasms. It shows positivity in the intestinal pattern component and is negative in the acinar component.

A.P results reported the following (Figures 1–5):

1. Gastric collision tumour. First component: moderately differentiated tubular papillary adenocarcinoma with invasion of the muscularis propria. Second component: acinar cell carcinoma with involvement of the muscularis propria. Both components are separated by a band of fibrous tissue and areas of necrosis, occupying 30% and 70%, respectively. No lymphovascular invasion is seen. Free surgical margins.
2. Type I heterotopic pancreatic tissue underlying the tumour. Acinar Focal CK7 (+), tubulopapillary CK20 (+), CD45 (-), chromogranin (-), synaptophysin 10%–15% and P53 (+) in both histological lines.

## Discussion

At the beginning of the 20th century, collision tumours located in the stomach were rarely reported; the first reports date back to the end of the 19th century by Dreyer (1894): fibrosarcoma/adenocarcinoma; Saar (1918): globocelular sarcoma/adenocarcinoma; Göting (1931) and Cornelius (1949): spindle cell sarcoma/adenocarcinoma; Battaglia (1951): lymphosarcoma/adenocarcinoma; and Stout (1953): rhabdomyosarcoma/adenocarcinoma. All of these were described in one of the first reviews by Wanke *et al* [2].

These tumours are highly infrequent and are rarely diagnosed preoperatively. The literature is scarce, and so is the information about them. A review of case series describes its predominance in males, as well as higher prevalence between the fifth and sixth decades of life [13]. Collision tumours must be differentiated from other entities such as carcinosarcomas (a single neoplasm that exhibits a carcinomatous and sarcomatous pattern), compound tumours (two different patterns intermingled in a single tumour) or from cancer to cancer metastasis (carcinoma that metastasises to another carcinoma) [12, 13].

Additionally, neoplasms with two different cellular populations but without a clear-cut interface between the histological patterns or with a mixed transition zone should be considered as composite and not as collision tumours [11–14]. Dodge [11] reported 7 cases of carcinomas of mixed aetiology in a series of 87 gastroesophageal carcinomas and pointed out that to describe a tumour of mixed structure as a collision tumour, there must be independent simultaneous growth of two neoplasms, with tumour areas separated in two distinct histological patterns and, if both tumours metastasise, the two types of growth should be clearly separated in the metastasis also. Furthermore, there should be no areas of transitional pattern between the two tumours [1].

Tumour genesis theories of these types of neoplasms do not satisfy all cases. The oldest theory is the accidental encounter of two neoplasms developing independently that eventually collide [12].

A second theory, known as the ‘common carcinogen’, suggests that a single carcinogen leads to the development of two adjacent synchronous neoplasms. The ‘common carcinogen’ theory is attractive as an explanation for the origin of gastric lymphoma/adenocarcinoma collision tumours. The relationship between pathogens, such as *Helicobacter Pylori* or Epstein–Barr virus (EBV) and the development of gastric lymphomas and adenocarcinomas, has been described (*H. pylori* is found in 45%–90% of the patients with gastric adenocarcinoma and in 56% of the patients with gastric lymphoma, while EBV has been found in 9%–16% of gastric adenocarcinomas and in 9%–16% of lymphoma patients) [12, 13, 15]. In addition, the association between *H. pylori* and the development of mucosa-associated lymphoid tissue (MALT) lymphomas is known, being the most frequent lymphoma among collision tumours [16]. Finally, there are other substances that have experimentally induced this phenomenon in rats, such as N-methyl-N-nitro-N-nitrosoguanidine associated with the development of adenocarcinoma/leiomyosarcoma [17].

On the other hand, the carcinogenic stimulation of one tumour over another tumour has been proposed as another possible theory. This theory states that one tumour facilitates the development of a second primary tumour. De Leval *et al* [18] described a case of collision of a gastrin-producing carcinoid tumour and a gastric adenocarcinoma. They proposed that the trophic effect of gastrin over gastric mucosa could induce the development of gastric adenocarcinoma. Also, Yanagawa *et al* [19] proposed that immunosuppression induced by a lymphoma could lead to the development of adenocarcinoma [13].

Additionally, clinical and experimental data have been reported, suggesting that at least one of these collision tumours originates from a single progenitor cell, which later differentiates into two different histological types. Milne *et al* [20] conducted a p53 and loss of heterozygosity (LOH) analysis in two collision tumours of the gastroesophageal junction and found that these tumours shared the same mutation in p53 and the same LOH pattern. Furthermore, Fukui *et al* [21] carried out the same experiment in a gastric collision tumour (neuroendocrine carcinoma/adenocarcinoma) and found the same p53 mutation and that the accumulation of the p53 mutation varied in each of the components [12, 13].

As stated earlier, a gastric collision tumour is an infrequent finding: lymphomas/adenocarcinomas (26 cases), gastrointestinal stromal tumours/adenocarcinomas (9 cases), squamous cell carcinomas/adenocarcinomas (7 cases), carcinoid/adenocarcinoma [18] among others. Each category presents unique characteristics, different behaviour and possible different origins [13]. During the literature search carried out for this case report, no description of a gastric tumour with an adenocarcinoma component in collision with an acinar cell carcinoma was found. Carcinoma/carcinoma cases are very rare.

On the other hand, there are reports regarding the malignant degeneration of a heterotopic pancreas (HP) of gastric location. HP was first reported in 1,727 by Jean-Schultz, who described this congenital abnormality. The most accepted genesis theory points out that during the development of normal pancreas from evaginations, originating from the wall of the primitive duodenal wall, one or more of these evaginations may remain in the intestine wall. The migration of this embryonic remnant along with the GI tract development would give rise to heterotopic pancreatic tissues [23]. HP can be classified according to Heinrich in 03 types: type I – containing acini, islets and ducts; type II – with acini and ducts; and type III – with ducts only. 90% of the cases of HP are found in the upper GI tract, with 25%–38% in the stomach, located in the submucosa, muscular or serous layer in 73%, 17% and 10%, respectively. It is generally located in the antrum (80%–95%), commonly in the greater curvature. Additionally, it is usually diagnosed between 50 and 60 years of age, with a higher incidence in men (3:1) [23, 24].

In the stomach, pancreatic tissue can be found in two different forms: as HP or as gastric pancreatic metaplasia characterised by nests, lobes or isolated pancreatic cells in gastric mucosa. The latter has been related with autoimmune gastritis [25, 26, 27].

Pancreatic carcinomas of exocrine or endocrine origin rarely develop in ectopic sites, where they can be misclassified as secondary tumours. Most of them are found in the stomach and tend to be contiguous with non-neoplastic exocrine pancreatic tissue.

Also, acinar cell pancreatic carcinoma is a rare neoplasm (1%) [25]. Up to 2009, only five cases of ectopic pancreatic acinar carcinomas had been reported, with three of them located in the stomach [25]. In 2017, Kim *et al* [28] conducted a review of six cases of heterotopic acinar pancreatic carcinoma in the stomach reported to date, highlighting the importance of immunohistochemistry (IHC). This report would be the seventh case of a pancreatic acinar carcinoma in the stomach and the first described in a collision tumour.

As stated before, IHC currently plays an important role in the diagnosis of HP and its malignancy, as well as in the distinction with neuroendocrine tumours. Markers described for pancreatic origin are positive for α-1 antitrypsin, chymotrypsin, alpha-1 antichymotrypsin, pan-cytokeratin and vimentin, and weak diffuse positivity for CD56 and DOG 1 clarify the diagnosis of HP. Also, markers necessary to distinguish from a neuroendocrine tumour are negative IHC for synaptophysin, chromogranin A, TTF-1, c-Kit, S-100 protein and CD34 [28]. Positive IHC for p53 has also been reported in about 30% of pancreatic acinar carcinomas [29]. Some of the markers mentioned before were obtained in this case report, stating that not all markers mentioned before are available in our pathology department, but its classification and differentiation from a neuroendocrine tumour was possible. It should be noted that in the reports, during the initial study for the diagnosis of these tumours, endoscopic biopsies were informed as poorly differentiated adenocarcinomas and in radiological studies were described as stromal tumours or lymphomas, which reinforces the need of IHC when the diagnosis is suspected or where doubt arises [29].

There is no consensus on diagnosis and treatment. Diagnosis is rarely made pre-operatively and, if done, there are two possible scenarios: the presence of both components in one of the samples or the presence of two different types of histological patterns in different samples, hence the importance of multiple biopsies of a suspicious tumour [13]. The definitive diagnosis of this type of neoplasm is carried out postoperatively by Pathology.

There are no specific signs or symptoms suggesting the presence of this type of tumour [13]. Apart from specific cases, such as the elevated gastrin level described by Yoshino *et al* [22], there are no laboratory data that suggest the presence of a gastric collision tumour.

Finally, due to scarce data and few long-term follow-up reports, it is logical to assume that surgery represents the treatment of resectable neoplasms, according to current treatment guidelines for gastric cancer. The indication of adjuvant therapy should be given according to the most advanced or aggressive histology pattern [13], strongly emphasising individualised and multidisciplinary treatment.

## Conclusion

In conclusion, this paper reports the first, to our knowledge, case of malignant gastric tumour with probable origin in heterotopic pancreatic tissue that collides with a gastric adenocarcinoma. A comprehensive review of the subject was made, emphasising the importance of the suspected diagnosis and joint work of the medical team for the final diagnosis of this rare entity.

## Conflicts of interest

The authors have no conflicts of interest.

## Funding

This research has not been funded.
